# Pearls and tips in coverage of the tibia after a high energy trauma

**DOI:** 10.4103/0019-5413.43376

**Published:** 2008

**Authors:** Antonio Rios-Luna, Homid Fahandezh-Saddi, Manuel Villanueva-Martínez, Antonio García López

**Affiliations:** Department of Orthopaedic, Hospital Virgen del Mar, Almería, Spain, and Neuroscience and Health Science Department, University of Almería, Spain; 1 Hospital De Alcorcón, Madrid, Spain; 2 IQTRA Medicina Avanzada, Madrid, Spain; 3 Hospital de Alicante, Alicante, Spain

**Keywords:** Open tibial fracture, soft-tissue damage, soft tissue coverage

## Abstract

Coverage of soft-tissue defects in the lower limbs, especially open tibial fractures, is currently a frequently done procedure because of the high incidence of high-energy trauma, which affects this location. The skilled orthopedic surgeon should be able to carry out an integral treatment of these lesions, which include not only the open reduction and internal fixation of the fracture fragments but also the management of complications such as local wound problems that may arise. There is a wide variety of muscular or pedicled flaps available for reconstruction of lower limb soft-tissue defects. These techniques are not commonly used by orthopedic surgeons because of the lack of familiarity with them and the potential for flap failure and problems derived from morbidity of the donor site. We present a coverage management update for orthopedic surgeons for complications after an open tibial fracture. We choose and describe the most adequate flap depending on the region injured and the reliable surgical procedure. For proximal third of the tibia, we use gastrocnemius muscle flap. Middle third of the tibia could be covered by soleus muscle flap. Distal third of the tibia could be reconstructed by sural flaps, lateral supramalleolar skin flap, and posterior tibial perforator flap. Free flaps can be used in all regions. We describe the advantages and disadvantages, pearls, and tips of every flap. The coverage of the tibia after a major injury constitutes a reliable and versatile technique that should form part of the therapeutic arsenal of all the orthopedic surgeons, facilitating the integral treatment of complex lower limb injuries with exposed defects.

## INTRODUCTION

One of the most important goals in the management of severe open injury of the lower limb is to obtain adequate soft-tissue coverage. It provides a close wound, to promote revascularization of injured bone and soft tissues, and to prevent late infections and nonunion that may occur secondary to persistent bone ischemia. Soft-tissue defects coverage of the lower limbs is currently a more frequent procedure due to the increase incidence of “high energy” traumas which affect this location.^1^–^4^ The skilled orthopedic surgeon should be able to carry out an integral treatment of these lesions, which include not only the open reduction and internal fixation of the fracture fragments but also the management of complications, which may arise such as local cutaneous wound problems. In some hospitals, there may be no plastic surgeon available on a daily or regular basis. Although some of the converging techniques or flaps may require the use of microsurgical instrumentation and skills with a special training, this should not be the case with the sural fasciocutaneous flap, gastrocnemius flap, soleus flap, and supramalleolar skin flap. The type of flap is chosen on the basis of anatomical considerations, specifically the location of the defect on the leg, the size of the defect, and the availability of local tissues for coverage.

The common denominator of this procedure is that the presumed healthy tissue proximate to the zone of injury is rotated on a vascular pedicle to provide coverage.

## PROXIMAL THIRD OF THE TIBIA

### Gastrocnemius muscle flap

It is very useful for treating defects that affect the proximal third tibia of the leg and anterior and medial region of the knee, such as the loss of substance after fractures.^5^–^7^ It is widely used because it is relatively easy to extract and has a constant blood supply. This flap can be used with muscle or with muscle and skin. It can also be pedicled (most common) or free.

**Figure 1:**
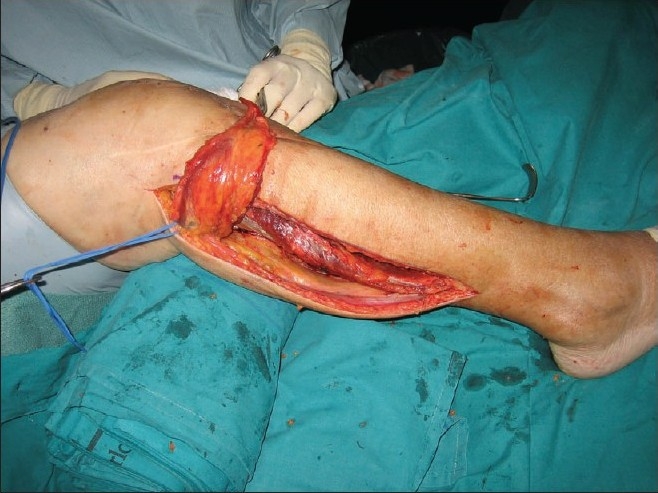
Preoperative photograph showing dissected lateral head of gastrocnemius flap

The medial head or the lateral head can be used. The medial head is more commonly used and is bigger than the lateral head, because the muscular body is smaller and the fibula constitutes an obstacle to passage of the flap to the knee. It is an excellent coverage of the lateral aspect of the metaphysis of the tibia^8^,^10^ [Figure 1]. The combination with a cutaneous paddle at the distal level of the muscle and over the distal aponeurosis makes it possible to use a bigger flap to cover part of the medial third tibia of the leg.

#### Indications

It is useful for the treatment of Grade IIIB open fractures that affect the proximal third of the tibia and for open fractures of the patella with loss of skin substance. It is also useful for the treatment of cutaneous problems after surgery on the proximal end of the tibia complicated by infection or a soft-tissue problem, and it can be used in atrophic pseudoarthrosis to improve blood supply in the area.^11^–^12^

#### Vascular supply

The flap used was Mathes-Nahai Type I.^5^ Both the medial and lateral head are irrigated by the sural arteries, which start in the popliteal artery and are accompanied by the motor nerve (tibial nerve). Each head can be moved individually on its own neurovascular pedicle.

#### Surgical procedure

The patient lay in supine position, with the limb in external rotation and the knee slightly flexed. The incision starts at midcalf, 2 cm behind the posteromedial border of the tibia and curves proximally to reach the popliteal fossa. It is important to avoid injury to the saphenous vein and nerve. The deep fascia is incised and retracted posteriorly as far as the plane between the two heads of gastrocnemius. A plane is developed between soleus and the medial head after the incision of the thin aponeurosis, and often the plantaris tendon is found in this space.

We dissect the sural nerve and retract it posteriorly. The space between the two heads of gastrocnemius is found. The distal tendon is divided, and the muscle is progressively raised in a distal to proximal direction. The motor nerve should be divided to avoid postoperative pain.

There are two tips to increase the arc of rotation: (1) to make multiple transverse incisions in the aponeurosis that lies on the deep aspect of the muscle and (2) to divide its proximal tendon insertion. The medial head isolated on its vascular pedicle, must be passed deep to the tendons of semitendinous and gracilis to reach the anterior aspect of the knee.

If we are using the lateral head flap, we divide the distal tendon and the transfer is progressively raised. The common peroneal nerve is mobilized, and the neurovascular pedicle is identified. If the muscle is needed to cover the anterior aspect of the knee, it will be passed deep to the peroneal nerve. This maneuver is not necessary when the defect is over the proximal third.

## MIDDLE THIRD OF THE TIBIA

### Soleus muscle flap

It is a very useful transfer for covering defects of the leg. It is frequently undamaged in compound fractures of the middle third of the tibia.^13^–^15^

#### Indications

The elective indication is coverage of the middle third tibia of the leg. If soleus is very long and its fibers insert on calcaneum, the transfer can cover the upper half of the distal third tibia of the leg. If there is a large defect but short in length, it is better to use a complete transfer of soleus. On the other hand, if it is narrow and extended along the tibial crest, we can use a medial hemisoleus transfer.

#### Vascular supply

The flap used was Type II in the classification of Mathes and Nahai.^5^ It is richly vascularized by the posterior tibial and peroneal arteries. Each artery gives a main pedicle proximally, an important pedicle at the midpoint, and smaller and variable pedicles along the distal third tibia. The entire muscle can survive only on its two main proximal pedicles, so the soleus can be mobilized without detaching its origin.

#### Surgical procedure

The patient is placed in supine position, with the limb in external rotation and the knee slightly flexed. The incision begins midway between the medial malleolus and the Achilles tendon and ascends as far as the proximal quarter of the leg 1 cm posterior to the medial border of the tibia. Deep fascia is incised preserving the saphenous nerve and vein. The border between soleus and gastrocnemius and the anterior border of the soleus is identified. The dissection between gastrocnemius and soleus is practically avascular. The intermuscular fascia between the two compartments should not be incised to protect the posterior tibial neurovascular bundle. We release the medial and the anterior aspect of the muscle. Midway between the medial malleolus and the knee joint, a large pedicle should be ligated.

The next step is to release the superficial aspect of the soleus distal to gastrocnemius with the finger and then release the soleus from the Achilles tendon. It is useful to retain a thin layer of aponeurosis. The final step is the release of the muscle from its lateral attachments. The lateral border must be released as proximally as possible to allow the rotation of the flap. The flap will cover the defects of the middle third tibia of the leg.^16^–^19^

## DISTAL THIRD OF THE TIBIA

### Sural flap

**Figure 2:**
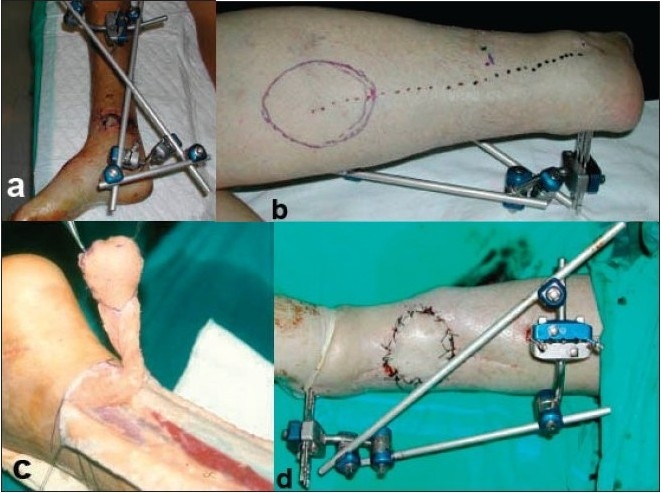
(a) Clinical photograph showing external fixator and soft tissue defect in open distal third tibial fracture. (b) Clinical photograph showing Sural flap marked out. We measured the defect to cover and based on its dimensions we draw the skin islet to be transferred in the form of a paddle centered over the depicted neurovascular sural bundle. (c) Preoperative photograph showing dissected sural flap. Dissection of the graft has to reach the distal limit which we marked, 4-5 cm proximal to the peroneal malleolus. (d) Clinical photograph showing skin defect, final closure.

In 1987, Ferreira *et al.* presented the concept of fasciocutaneous flap of the distal pedicle based on the inframalleolar perforators. In 1992, Masquelet *et al.* described the use of the neurocutaneous flap for the reconstruction of soft-tissue defects of the distal third tibia of the leg.^10^ Among the main indications for a sural fasciocutaneous flap are the soft-tissue defects of the heel and the external or internal perimalleolar regions [Figure 2a]. The advantages of the sural flap as compared with other covering methods are the simplicity of the design, dissection of the pedicle flap that can be carried out with a loup magnification and without the need of microsurgical instrumentation or anastomosis, the preservation of the principle vascularization of the lower limb, and the need for only one operation. The sural pedicled flap constitutes a well-vascularized cutaneous islet and reliable flap offering the possibility of covering a broad range of areas. The sural flap has been used in patients with diabetes mellitus and recurrent plantar ulcers that require a major muscular coverage.^20^–^23^

#### Indications

The indications are soft tissue defects in the distal tibia, heel, and “up to the” rear foot.

#### Vascular supply

The graft is based specifically on the vascularization that runs along with the sural nerve. Flap irrigation is accomplished by a distal reverse flow of the superficial sural artery dependent on perforators of the peroneal arterial system.

#### Surgical procedure

We used spinal anesthesia, and the patients were placed in prone position. A tourniquet is applied in the mid thigh, and exsanguination was achieved by the elevation of the lower extremity that facilitates the identification and dissection of the neurovascular structures in contrast with the use of the Esmarch bandage [Figure 2b].

The sural fasciocutaneous flap (SFF) is made up of skin and subcutaneous fat, the superficial and deep fascia of the posterior part of the leg, sural nerve, the sural vein, and the superficial sural artery. There are numerous anastomoses between the peroneal artery and the vascular axis of the flap. The most distal is found usually at a distance of 4-5 cm above the tip of the lateral malleolus, and thus it is taken as the point where the pedicle pivots. The sural nerve is supplied by fasciocutaneous branches of the peroneal artery in the distal two thirds of the leg. The proximal third is supplied by superficial sural artery. Some authors perform a Doppler test before the intervention to confirm the integrity of the peroneal artery, its anastomosis with the fasciocutaneous branches, and the precise localization of the pivot point on which the flap should rotate. Other surgeons perform a Doppler examination of the lower limb only when there is reasonable doubt as to the normality of the peroneal artery, which is the anatomical basis of this graft.

Proximally, this flap cannot be delineated further than the junction of the heads of the gastrocnemius, as at this level the sural nerve and the artery are subfascial until they reach the popliteal fossa.

We have always initially drawn the pivotal point of the pedicle 4-5 cm from the peroneal malleolar tip. We outlined the expected trajectory of the sural nerve, artery, and vein in the posterior aspect of the calf. Then we measured the defect to cover, and on the basis of its dimensions, we drew the skin islet to be transferred in the form of a paddle centered over the depicted neurovascular sural bundle. A broad dissection was carried out to incorporate the deep fascia, dividing the sural nerve proximally and ligating the artery and the vein. The entire flap was elevated and dissected from proximal to distal. Stay stitches were placed between the skin and subcutaneous fascia to prevent sliding of both layers that may damage the perforators, which constitute the basis of this flap. At this stage, careful diathermy of the bleeding perforators in the lateral margins of the flap was mandatory. In this way, the gastrocnemius musculature was left exposed. Dissection of the graft had to reach the distal limit that we marked, i.e., 4-5 cm proximal to the peroneal malleolus. The width of the carrier pedicle was 3-4 cm. A subcutaneous tunnel was created, and the flap was then transposed to the area of the defect we wished to cover, by carefully rotating the flap over its pedicle up to 180° [Figure 2c].^24^–^26^

The donor site was closed with interrupted sutures if the islet skin flap dissected was no wider that 5-6 cm. Otherwise, a free skin graft may be used to cover the defect created. At the end of this procedure, the tourniquet cuff was deflated, and the adequate circulation of the flap verified. Numerous punctures of the flap, using a 25-G needle, were made to allow bleeding, thus minimizing hyperemia and venous congestion [Figure 2d]. A drain was left under the rotated flap to minimize the likelihood of hematoma formation. The limb was dressed with cotton and elastic bandages. The largest size of the flap documented in the literature is 17 × 16 cm, but the complication rate increases accordingly with the graft size. With enlarged flaps, the larger pedicle may be compressed more easily once it is tunneled and post-operative swelling increases, which may augment venous congestion of the flap with the risk that this involves and a greater possibility of suffering partial necrosis of the skin bridge under which the graft is tunneled. Other authors do not tunnel the flap under the skin because of the fear of compression of the fatty pedicle against the skin especially in the postoperative phase when more swelling develops. The donor area morbidity increases in relation to the size of the flap dissected to cover the defect created with a large free skin graft.

### Lateral supramalleolar skin flap

The lateral supramalleolar flap was described in 1988, thereby expanding the armamentarium of locoregional flaps for coverage of the ankle and foot. Reconstruction of these areas is often a tough challenge for plastic surgeons because of the location of the skin defect and the vascular condition.^27^–^29^

Lateral supramalleolar skin flap offers a range of coverage similar to that of the sural flap, but the dissection is more difficult than sural flap and offers no advantages over it; the area of anesthesia in sural flaps is smaller than after transecting the superficial peroneal nerve. Theoretically, the sural flap does not cover as distally as the supramalleolar flap, but some authors testified that the distally based sural flap is more reliable than the lateral supramalleolar flap, especially regarding the venous congestion and have shown the usefulness of the sural flap for weight-bearing areas even when resensibilization is not performed; lateral supramalleolar skin flap is not recommended as a coverage in this areas. The global proportion of failures is almost four times greater when compared with that for the supramalleolar skin flap.^14^

#### Indications

Indications are the soft tissue coverage of lower leg, ankle, and foot skin defects. Coverage of the weight-bearing surface of the foot should be avoided.

#### Vascular supply

The premalleolar region is over an anastomotic junction between the anterolateral malleolar artery (branch of the anterior tibial artery) and the anterior branch of the peroneal artery. Multiple anatomic variations determine the technique of flap elevation. In its classic form, the flap, vascularized by a cutaneous branch arising from the perforating ramus of the peroneal artery, is raised on a retrograde vascular flow. The peroneal artery, in its distal course behind the tibiofibular angle, provides a perforating branch that traverses the interosseous membrane. This branch provides multiple ascending branches to the overlying skin.

#### Surgical procedure

**Figure 3:**
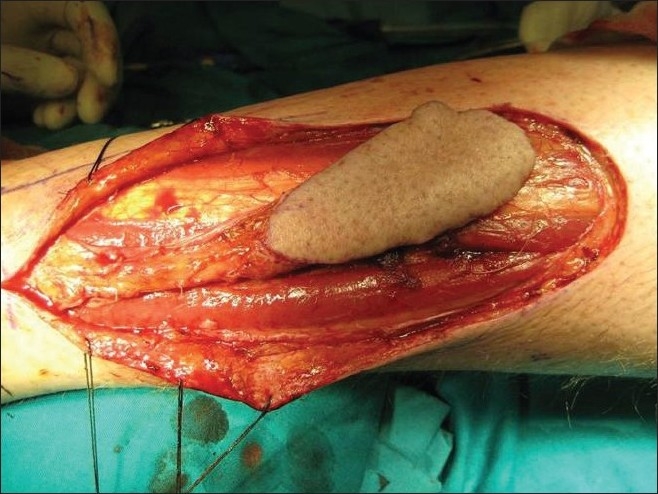
Preoperative photograph showing dissected lateral supramalleolar skin flap

In its usual form, the flap extends from the tibial crest anteriorly to the posterior border of the fibula laterally. Its distal limit must include the depression between the tibia and the fibula, which is a constant surface-marking for the site of emergence of the perforating septocutaneous branch of the peroneal artery. The outline of the flap must include this landmark, being traced 2-3 cm distal to it. The skin is incised along the anterior margin of the lateral malleolus. The pedicle is deep to the superior extensor retinaculum that is incised. The pedicle is exposed over the anterior tibiofibular ligament. Once the pedicle is visible, the rest of the skin paddle is elevated (which necessarily includes division of the superficial peroneal nerve) [Figure 3]. If a longer pedicle is wanted, the transverse anterolateral malleolar artery (a branch of the anterior tibial artery) is ligated. The flap is then vascularized exclusively by the anastomotic arcades in the foot. The flap can also be raised on an anterograde-flow vascularization by ligature of the anterolateral malleolar artery beyond its anastomosis with the peroneal perforator. This latter artery is also ligated, and the flap is then pedicled on the anterior tibial artery. The maximal length of the vascular pedicle is 7-8 cm, which allows this flap to be used to cover skin defects over the medial aspect of the leg, ankle, and forefoot. The posterior margin of the flap is incised including the fascia and reflected anteriorly, thus exposing the peroneal muscles. At this stage, the flap remains attached only to the septum that separates the anterior and the lateral compartment. This septum is divided close to the fibula, and the flap is raised. It is important to divide the posterior border of the fascia of extensor digitorum brevis to avoid compression of the pedicle.

### Free flaps

Very often, the best solution for defects of the medial-distal third of the tibia is microvascular transplantation. During the last 30 years, the development of microsurgery techniques and their application to the reconstruction of the lower limbs make free flaps the technique of choice, especially for large defects or combined defects. The technique is indicated for high-energy trauma, actinic lesions, osteomyelitis, and recalcitrant pseudoarthritis. Of the more than 60 free flaps described, the most useful for covering defects of the medial and lower third of the leg are muscular flaps such as those from the rectus abdominis, latissimus dorsi, serratus, and gracilis muscle, and fasciocutaneous flaps such as scapular, parascapular, and anterolateral thigh flaps.^30^

**Figure 4:**
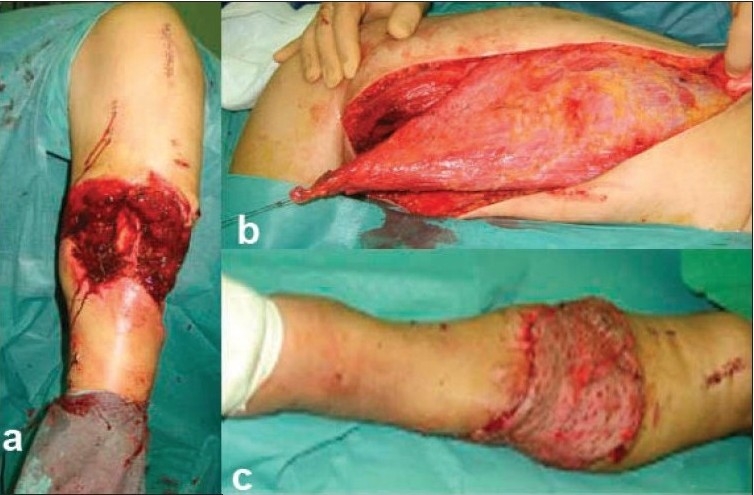
(a) Clinical photograph showing middle third open tibial fracture with big soft tissue defect. (b) Preoperative photograph showing latissimus dorsi muscle free flap, proximally based at the donor site. (c) Clinical photograph showing final coverage of defect.

Muscular flaps share characteristics that make them ideal for the reconstruction of the lower extremity—they can be molded and adapted to the irregular geometry of the wound [Figure 4a]. Their large volume makes them ideal for filling large defects if they are transferred free. They have a rich blood supply, and this is an advantage considering the ischemia that is normally associated with posttraumatic defects, infected tissue, or irradiated tissue of the lower limbs. The contour of muscle flaps improves over time, unlike that of fasciocutaneous flaps, which can become edematous or congestive in certain positions. The esthetic result with a muscle flap is acceptable, although it is inferior to a fasciocutaneous flap. Morbidity of the donor area is low when a large amount of tissue is necessary. Lastly, all the different types provide a long vascular pedicle, although the only drainage source is the vein of the main pedicle. Another disadvantage is that the area with the flap can be intolerant to cold, as it does not have subcutaneous tissue.

### Latissimus dorsi muscle flap

This is probably the most employed and reliable free flap in the whole body.

#### Indications

Indication is the soft tissue coverage of large defects of the leg.

Advantages of the latissimus dorsi muscle flap include the following: Large volume of tissue is available for reconstruction, long vascular pedicle offers excellent range for pedicled flaps, high caliber pedicle makes free flap vascular anastomoses technically more feasible, even in patients with significant atherosclerotic disease; independent skin paddles can address complex defects, minimal donor site morbidity occurs and it can be combined with other subscapular flaps, when indicated.

#### Vascular supply

The latissimus dorsi muscle is supplied by two separate vascular systems. The dominant blood supply arises from the thoracodorsal artery, which is the terminal branch of the subscapular artery. It also has a secondary blood supply, which arises from segmental perforating branches off of the intercoastal and lumbar arteries. These vessels enter the deep surface of the muscle near the posterior midline and are responsible for perfusion of the inferior and medial latissimus. Because these vessels are disrupted in the process of harvesting the latissimus, the viability of this portion the flap can be tenuous.

The thoracodorsal artery and vein course along the thoracic wall on the undersurface of the latissimus muscle. Overall, the extramuscular pedicle length varies between 6 and 16 cm and is about 9 cm on average.

#### Innervation

The motor nerve to the latissimus dorsi muscle is the thoracodorsal nerve, which arises from the posterior cord of the brachial plexus and is derived from the sixth, seventh, and eighth cervical nerve roots. The nerve travels distally with the vascular pedicle and supplies only the latissimus dorsi muscle.

#### Surgical procedure

The patient lies in the midlateral position or supine with a sandbag. A longitudinal incision is made from the axilla to the posterior iliac crest. We always mark out the skin paddle required.

Then, the lateral border of the muscle is exposed. The key point of the technique is the anterior border dissection to expose the vascular pedicle. As a free flap, the pedicle must be carefully dissected, and the muscle should be released from its spinal origin and from the iliac crest. We should carefully ligate the small vessels deep to teres major. The scapular insertion is divided too. The segmental vascular intramuscular division allows to split the muscle and to raise the medial portion only [Figure 4b and c].

## DISCUSSION

There is a wide variety of muscular or pedicled flaps for reconstruction of lower limb soft-tissue defects. The use of these techniques is not common among orthopedic surgeons due to the lack of familiarization with them and the potential for flap failure and problems derived from morbidity of the donor site. Free flaps, lateral supramalleolar skin flap, posterior tibial perforator flap, and sural flaps are the most common flaps for coverage of the tibia.^1^–^3^,^32^,^33^

The gastrocnemius muscle is the preferred muscle for coverage of the knee, and either the medial or lateral head can be used. Use of the medial head is preferred because it is larger and more mobile and thus easier to conform to different size and shape defects. Use of the medial head of the gastrocnemius muscle does not result in significant loss of function because of the remaining soleus and lateral gastrocnemius.

Transplantation of the soleus muscle into medial and distal third leg wounds was first described clearly by Wright and Watkins. When the soleus muscle is undamaged and suitable technically for coverage of a defect, it should be used. When it is feasible to use the proximal soleus muscle, it provides a very simple solution to some difficult lower tibial wounds that would ordinarily be treated with a so-called free flap. In all these patients, the feasibility of soleus flap coverage was determined during operative exploration. Further consideration of soleus flap use in this area, however, may lead to the desirability of preoperative assessment of the condition of the soleus muscle in a traumatized leg. Conventional arteriography and magnetic resonance imaging could yield pictures of the segmental blood supply of the soleus, and the latter could show details of the muscle structure. With wider application of soleus flap coverage, such preoperative studies may prove useful.

The advantages of the sural flap as compared to other covering methods are the simplicity of the design and dissection of the pedicle flap that can be carried out with a loop magnification and without the need for microsurgical instrumentation or anastomosis, the preservation of the principle vascularization of the lower limb and the need for only one operation. The sural pedicled flap constitutes a well vascularized cutaneous islet and reliable flap offering the possibility of covering a broad range of areas with cutaneous defects in the distal tibia, heel, and up to the rear foot. The sural flap can be especially useful in cases of injuries with serious compromise to the circulation of the major arteries of the lower limb as long as the peroneal artery is intact or when a microsurgical procedure is contraindicated. One of the few disadvantages of this flap is that by sacrificing the sural nerve, an inevitable anesthesia area appears over the lateral aspect of foot, which is usually well tolerated by the patient. The survival index of the flap exceeds 90%. It can be used in an emergency situation, and it does not expose it to the failure of the flap.^32^

Free flap reconstruction of defects requires lengthy costly hospitalization, microsurgical training and experience, special instruments, and a two-team approach. The long operative time and functional donor-site morbidity are major disadvantages of this method. Good planning and microsurgical experience are the most important factors for successful results. Free flaps are advised for extensive skin defects or in cases in which poor distal vascularity of the leg or local trauma precludes the reliability of a distally based fasciocutaneous flap. The success of free flaps on the lower limbs depends on the same basic principles as reconstruction with local flaps, that is, adequate debridement, excision of necrotic tissue and avascular bone, rigid bone fixing of unstable bone fragments, and vascular microanastomosis to suitable recipient vessels. To achieve the greatest flow possible, arterial suture to the most available proximal and largest-caliber vessel (end-to-side) is recommended. Flow should be anterograde, and the sutures should be end-to-side to improve the entry of blood into the vessel—this reduces the formation of thrombi and does not sacrifice a larger blood supply. There is general agreement that the suture should be as far away as possible from the so-called lesion area, which led Acland to coin the term “posttraumatic vascular disease.” This vascular disease is especially evident in infected pseudoarthrosis, even in apparently healthy areas. Therefore, the suture should be at least 7-9 cm away from the lesion, and the compartment should be changed if possible. For example, in a defect involving the medial third of the tibia, the popliteal vessels should be used. Of the three arteries in the leg, the peroneal is too deep and the most difficult to access and the tibial artery in the anterior compartment that is closest to the bone is usually more affected by posttraumatic vascular disease, especially in chronic situations. Therefore, we prefer the popliteal vessels or the posterior tibial artery as recipients in end-to-side suture to avoid eliminating a major blood supply in the leg. However, this is a controversial topic, because some authors recommend end-to-end sutures as they are safer when there is previous vascular surgery or thromboembolic disease. When the recipient artery is the posterior tibial, it is better to use the end-to-side approach to preserve the arterial axis. In the case of the anterior tibial artery, the end-to-end or end-to-side approach can be used if there is an important difference in caliber. The recipient vein is also important to ensure correct drainage of the flap. The ideal recipient vein should be anterior to avoid compression in the decubitus position, and it must have a constant caliber, be easily dissected, and be situated close to the area of the arterial anastomosis. The saphenous vein meets all these requirements. In addition to the internal saphenous vein, the anterior tibial, posterior tibial, external saphenous, and even the popliteal vein can be used.

If suitable proximal suture is not possible, two maneuvers can be used: vascular bridges with vein grafts at the same time as the flap, or the initial use of a saphenous loop to provide healthy recipient vessels close to the lesion with a large vascular flow followed by fixing of the flap. It is important to bear in mind the caliber and length of the pedicle for each flap and its hemodynamic characteristics, which will make it more or less tolerant to a vascular bridge flap.^1^,^4^,^7^,^8^

When surgery is elective, a preoperative arteriogram is useful, as it determines the presence of distal or proximal atherosclerotic disease, tells us the state and permeability of the distal trunks, indicates the presence of posttraumatic fistulas, and gives us an idea of the state of the vessels in the distal area of the lesion, which is easier to access and more comfortable for suturing. Arteriography should never replace intraoperative analysis of the vessels.
